# Mitigating risks of students use of study drugs through understanding motivations for use and applying harm reduction theory: a literature review

**DOI:** 10.1186/s12954-017-0194-6

**Published:** 2017-10-06

**Authors:** Dor David Abelman

**Affiliations:** School of Health Studies, Faculty of Health Sciences, Western University, London, ON N6A 5B9 Canada

**Keywords:** Substance-related disorders, Study drugs, Postsecondary education, Harm reduction, Self-medication hypothesis, Qualitative research, Primary prevention, Methylphenidate, Adderall

## Abstract

As postsecondary students’ use of “study drugs” becomes more popular with increasingly reported negative effects on health and academic performance, failing prohibitionist policies to reduce consumption, and ambiguity in literature towards best practices to address this population, we present a literature review that seeks effective solutions educational institutions can apply to improve outcomes for students who use drugs. Motivations for use, effects of the substances, an analysis of efforts to control use from educational institutions, and suggestions on promoting most effective outcomes based on harm reduction, are described. Theory, quantitative, and qualitative works from systematic reviews, cohort studies, and epidemiological assessments are examined on the “study drugs” methylphenidate, dextroamphetamine, and amphetamine, also known as Adderall, Ritalin, Focalin, and Concerta. There is a focus on postsecondary students ages 18–25 in North America. Results show important risk factors for drug use including low perceived self-efficacy or enjoyment in courses, poor accommodation of special needs, reliance on external validation, having a low GPA, and experiencing a mental health issue. There is much misconception on the health and academic effects of these drugs in literature, among students, and on online knowledge sources. We suggest these drugs do not improve GPA and learning, while they might temporarily increase memory, but with detrimental negative health effects. Campaigns that address underlying factors of use can be most successful in mitigating harms.

## Background

In their interesting literature review, Bostrom and Sandberg (2009) define cognitive enhancement as using artificial means to optimize one’s learning and memory systems [1]. Some methods of enhancement include genetic modifications, mental training, brain-computer interfaces, and pharmaceutical substances [1]. In Smith and Farah’s descriptive systematic review, the categories of pharmaceutical substances are narrowed into a group of “study drugs” [2]. It is here the authors introduce the reader to derivatives of methylphenidate, better known as Concerta or Ritalin, dexmethylphenidate, better known as Focalin, and a combination of dexmethylphenidate and amphetamine, better known as Adderall [2].

Smith and Farah discuss the drugs in detail; methylphenidate, dextroamphetamine, and amphetamine are stimulants that inhibit dopamine and norepinephrine reuptake in the brain [2]. These neurotransmitters play an important role in cognition and pleasure [2]. People with attention deficit (hyperactivity) disorder, ADD/ADHD, are believed to have an imbalance of these neurotransmitters that the drugs help restore [2]. The off-counter use of these four drugs for studying purposes by university and college students ages 18–25, without attention deficit hyperactivity disorder (ADHD), in North America, will be the focus of this paper. Usage of these drugs by populations who are prescribed it and in other regions of the world will also be considered.

While there is highly mixed evidence on if the drugs can improve learning, working memory, and cognitive control in healthy individuals (with most studies suggesting they do not), many students believe they are effective aids to completing academic coursework [2–8]. Some authors believe that the drugs may increase the perception of work accomplished, energy, and motivation, but that actual cognitive ability remains unchanged [2, 6]. Other studies suggest there may be a mild improvement in learning and memory, but stronger studies are recommended to confirm these effects, that side effects health effects may occur, and that the effects do not increase grade point average (GPA) [2, 5–8].

A further review of literature shows that approximately one third of studies show null results on the effects of methylphenidate, dextroamphetamine, and amphetamine on learning [2]. There may be more studies with null results that are unpublished due to publication bias favoring significant results [2]. Some studies show some improvement in consolidation of long-term declarative memory, although there is no consensus in literature on this, with many cases of individuals performing worse or experiencing detrimental health issues due to the drugs [2, 3, 6, 8]. While memory may be improved over the short term, long-term effects of these drugs and the sleep deprivation they may cause may outweigh their positive memory improving effects [6]. It appears that the negative cognitive effects are highest among students who already perform highly [3].

Medication guides, the American Addictions Centers, and research on how the brain responds to drugs characterize stimulants such as Adderall, Ritalin, and Concerta as one of the most addictive substances at high doses that should be dispensed sparingly [9–11]. They can cause severe and dangerous addiction that can lead to sudden death, toxic psychosis, anxiety, sleep disturbances, and cardiac issues [9–11]. These effects are noticed among university students; a comprehensive literature review describes that increased rates of non-prescribed use of these substances can be directly correlated to an exponential increase in related suicides, emergency room visits, and dangerous overdoses, by students in recognized well-established universities in North America [12]. Furthermore, neuroscientists can determine how these drugs physically deteriorate brain processing ability in healthy individuals through tracking cognitive activity with radioactively labeled glucose [8]. It is important to note, however, that the United States Food and Drug Administration reports theraputic doses of these drugs in individuals who are diagnosed with ADHD are substantially safer [13, 14]. When used at low doses as prescribed to treat symptoms of ADHD, there is no evidence that using these drugs significantly increases risk of a severe cardiac event from occuring, although a mild increase in risk is observed [13–15].

## Contemporary issues

Prevalence of study drug use is increasing. By the end of 2009, at least 25 studies reported surveys of non-medical “study drug” use among college students [2]. Total percent of the student population who used study drugs in the past year, measured through self-administered surveys mailed to the students, ranges from 4.1 to 11.2% in the USA (where there is more national prevalence data collected) and approximately 8% in Canada [16–19]. When asked if they ever tried unmedicated study drugs in their lifetime, the number is higher from approximately 12% as a US national average, 15% among Canadian medical students, and even 55% in a group of fraternity students [16, 19, 20]. Most who use these medications receive them from friends who are diagnosed with ADHD [2]. Finally, the rates of study drug use are steadily increasing in universities throughout North America [4, 21].

This prevalence increase is problematic as use of study drugs can pose significant health issues. The US National Institute on Drug Abuse shares that study drugs can produce symptoms of malnutrition and high blood pressure, feelings of anxiety, and increase the likelihood of experiencing a stroke [22]. The drugs rapid increase of dopamine in the brain can disrupt normal communication between brain cells and increase the risk of addiction [9, 2]. Pharmaceutical companies and addiction associations describe them as highly addicting substances with the potential to cause dangerous cardiovascular complications including sudden cardiac death, stroke, and seizures, as well as problems in other organs including the liver and kidneys [10, 11]. They have also been shown to induce states of psychosis, paranoia, anxiety, and depression in some individuals [9–11].

Following these negative health issues, some prestigious universities believe the negative effects of study drugs outweigh the positive [23]. Some of the actions they take to reduce study drug use are controversial and will now be discussed. Duke University banned the use of “study drugs” as academic dishonesty with severe consequences up to expulsion [23]. To protect students, other large universities in North America banned college clinicians from diagnosing ADHD or prescribing related medications, forced students with ADHD to sign contracts that they would not share their medication, or included study drug use education sessions in their orientation week [12].

The efficacy of addressing study drugs as an issue of academic integrity following Duke University’s example is highly debated [23–25]. Maahs’ new article based on a survey of students in a well-established university found that personality characteristics of general deviance were the most significant risk factor predicting a student’s use of study drugs [24]. According to this theory, students use study drugs to rebel from authority. The more aware students are of a prohibition towards study drug use, the more likely they are to use these drugs [24]. Another article based in a similar region, time, design, and sample population suggests the opposite. The article finds a student’s belief that using study drugs is wrong was among the strongest factors that decreased their likelihood of use. The article directly encourages academic policies against study drugs be employed to reduce their consumption [25].

Lastly, the idea that prohibiting drugs is not the most effective method of reducing their use, as in the case of Maahs’ article, has also been found in a variety of research related to other drugs around the world [26, 27]. A new report from the *Lancet Commission on Drug Policy and Health* challenges prohibiting drugs with strong arguments related to health, human rights, economics, and international development [27]. They cite regions such as Portugal, Switzerland, the Czech Republic, and a city in Canada as case examples [27]. Studies in neuroscience show that simply prohibiting the use of drugs and describing them as simple control issues in which users can naturally stop does not support best models of recovery from drug addiction [28]. Solutions with stronger evidence yielding clearer and more positive recommendations for decreasing study drug use will now be discussed.

## Discovering real motivations for drug use

Arria and Dupont (2010) discuss in their literature review how decreasing study drug use, such as through educational sessions in orientation weeks, is gaining attention and positive support as new research helps understand why students use study drugs in the first place [3]. Main reasons for use include misinformation on the effects of these drugs on health and academic improvement and the use of study drugs as coping mechanisms for deeper underlying issues [3, 4]. It is important to note that not all students who use these drugs may be experiencing a deeper underlying issue, as many students may use it recreationally and function normally, but important to consider this risk group to help promote effective solutions for those who do [2].

Many students believe that study drugs help improve their grade point average (GPA), and this belief increases their risk of using them [3]. This view is challenged by a recent rigorous longitudinal cohort study found that non-prescribed prescription stimulants had no significant effect on university GPA [5]. Furthermore, research suggests that having a low GPA is a significant risk factor for using study drugs [12, 29]. Cohort studies support that underlying issues can be associated with low GPA by demonstrating how non-academic factors such as high parental monitoring, task solving skills, participating in a sports team, and perceived self-efficacy all help to improve and predict university student’s GPA [30–33].

The findings that prescription stimulants commonly do not improve academic performance are consistent with other research that suggests they may be more of a means of coping for a deeper underlying issue [34]. In addiction science, this coping is recognized as Khantzian’s ‘Self-Medication Hypothesis’ [35]. This hypothesis asserts that an individual uses a drug to achieve a particular emotional state which the substance promotes [35]. Common motivations for achieving such a state are to address feelings of stress, feeling overwhelmed, or of low self-esteem, and to promote feelings of confidence, calmness, and being in control [35]. When an individual becomes reliant on a substance to promote these feelings, symptoms of addiction follow [35]. This model of motivations for drug use can be shown through qualitative interviews and quantitative surveys [1, 34, 36, 37].

Vrecko’s qualitative interviews with university students who use study drugs describe that these drugs are primarily an emotional coping mechanism to address a lack of confidence, enjoyment, and interest in their coursework [34]. Giordano’s survey of 3038 students shows that those who feel reliant on external validation of their self-worth are significantly more likely to use study drugs than those who are less reliant on others opinions to feel confident [36]. Bostrom and Sandburg’s review described the use of prescription stimulants as coping mechanisms for the failure of educational institutions to meet the needs of students with special learning requirements who may struggle academically [1].

Bostrom’s findings show similar results to Currie’s (2013) review, which suggests that since policy changes made it easier for Canadian children to be diagnosed with ADHD, these drugs may not even help improve outcomes for those who are prescribed them [37]. Increased access to the drug methylphenidate (Ritalin) actually increased the probability of the students with ADHD not completing a postsecondary education, committing minor crimes, and girls showed an increased likelihood of experiencing depression [37]. The authors attribute social factors such as the stigma of having ADHD, as well as personal factors such as having an underlying behavioral issue requiring care beyond medication that is not provided in an educational setting, as motivations for negative health outcomes [37]. Medication has been very effective, however, in reducing symptoms for many individuals who have ADHD in other works [7, 14, 38–40]. The potential issues of worse outcomes in Currie’a work may arise in populations who are misdiagnosed, as users of these medications without ADHD tend to have the worse outcomes than those who are prescribed them for symptom management, and the rate of young adults diagnosed with ADHD is increasing signficicantly [3, 37, 41].

Another important risk factor for abusing a prescription stimulant drug is dealing with an underlying mental health issue [3, 42]. Ward, Oswald, and Galante’s (2016) team found off-counter use of prescription stimulants was a risk factor for alcohol abuse and eating disorders among students in a large North American university [42]. This suggests it might be a coping mechanism to deal with risk factors of those illnesses such as anxiety [42]. The strong influence of anxiety, self-efficacy, and self-awareness on these disorders are discussed extensively in the new results of Micali et al.’s (2017) analysis of a cohort study of approximately 15,000 children who were followed from birth [43]. This suggests that some students are using the drugs to deal with underlying symptoms of mental health issues, just like as described Khantzian’s self-medication hypothesis model on motivations for drug use [35]. Furthermore, individuals with previous history of mental health issues tend to have the poorest outcomes when using these substances [10, 11, 14].

As underlying risk factors for use have been identified, and current prohibitionist policies have been found ineffective for many populations, solutions to reducing a complex behavior such as study drug consumption could therefore be more effective if they focus on underlying risk factors for their use (such as low perceived self-efficacy, accommodating special needs, non-academic influences that contribute to a reduced GPA, and treating symptoms of an underlying issue) [1, 3, 24, 27, 34, 37, 42, 44]. Solutions applied in practice will now be suggested and evaluated from a harm reduction perspective.

## Solutions from harm reduction and addiction science

In their comprehensive review of factors influencing risk of study drug use in North American universities, Arria and Dupont (2010) directly suggest study drug users should meet with mental health professionals [3]. They describe how study drug use can be a significant ‘red flag’ of underlying mental health issues [3]. The concept that people can continue to use drugs while taking precautions to mitigate risk, but not necessarily decreasing their consumption, relates to harm reduction [45]. Harm reduction is based on the recognition that many people will continue to use drugs through efforts to prevent their use, that the majority of them do not need treatment, and that there are things that can be done to help these groups achieve healthier and more favorable outcomes [45]. It focuses on principles of public health and human rights that are practical, feasible, and effective [45]. There is a need to disseminate harm reduction information to help individuals and policy makers be in a position to make better-informed decisions relating to drug use and to provide more opportunities to lead healthier lives.

In the context of Arria and Dupont’s argument, encouraging a student who uses study drugs to feel more comfortable or aware of the availability of a mental health service is an example of harm reduction because it does not necessarily encourage the student to reduce their use, but continue to consume in a safer environment [3, 45]. Another harm reduction strategy Arria suggests is to provide systems in place that reduce peer pressure and stigma promoting a student with ADHD to share their medication, which has been reported to be high on university campuses [3]. This is important and concerning as students with ADHD are the main source of access for other students to reach these drugs, and it is reported that many do not adhere to taking thier medication regularly as prescribed [2, 3, 38]. The authors recommend that physicians and parents are key figures who can successfully empower students with ADHD not to share their medication and should have discussions with them on this topic [3]. Doing so can help students with access to this medication feel more comfortable using it safely for its intended purpose.

As some students with ADHD who used methylphenidate (Ritalin) had negative impacts on health and academic performance, potentially due to stigma associated with having ADHD, educational institutions may be able to improve outcomes for this population through improving inclusivity and integration between all students under their care [37]. Reducing stigma even among drug users without ADHD has been directly recommended to improve outcomes in other studies [3, 27]. In addition, as inability to accommodate a student’s special needs increased both risk of using drugs and the likelihood of experiencing worse outcomes from using them, implementing programs that help accommodate these students may be effective in decreasing overall consumption of drugs and improving outcomes for those who do [1, 37]. These outcomes are important components of harm reduction [45].

The findings of Vrecko and Giordano’s data, which suggests feelings of low perceived self-efficacy, little enjoyment in coursework, and strong reliance on external validity to establish self-worth were important components of drug use risk, adds a new potential site to focus on in efforts to reduce overall drug consumption [34, 36]. Campaigns that address these issues such as through working towards improving confidence, increasing self-efficacy, and making coursework more enjoyable, may promote better outcomes for this population [34, 36]. Furthermore, campaigns that promote high self-efficacy, (along with participating in a sports team, increased parental monitoring, and practicing task-solving skills) may also decrease overall drug consumption and mitigate harms of using them, such as by reducing overdoses, through increasing likelihood of maintaining a high GPA [12, 29–33]. This decreases the risk of using these drugs [12, 29].

Another way to mitigate risk addresses a reported a lack of knowledge on risks of using study drugs among the general student body [2–4, 12]. Universities can include a general education session on the dangers of study drug use, risk factors for use, and options to get help if already using for all incoming students during orientation week [3]. Doing so could help students who are currently using these drugs to learn where to get help to use them in safer ways, all students better understand some risk factors for using them and where to get help if they think they fall into a risk factor category, and discourage students interested in trying for the first time from putting themselves at risk or expose them to potential addiction [3]. This is similar to a survey finding students perceptions of high peer use of study drugs is a significant risk factor for their personal use [25]. If students feel that there are less people using these drugs as there are more options for alternative methods of support, drug use may decrease as a whole and existing use may be done in safer ways.

Social support, empowerment, and lack of knowledge are further shown to reduce study drug use in Holloway et al.’s (2013) review on factors influencing risk of using study drugs [4]. The authors searched five online databases to discover that student’s misunderstandings on the dangers of study drug use were prevalent partially due to false information on websites such as blogs [4]. It was recommended that physicians, pharmaceutical companies, and educational institutions be aware of this issue and educate university students on the true issues of using these drugs and ways to get help to reduce use [4]. Efforts to improve validity of drug-related information online is also important as Kam’s longitudinal study of 2749 youth finds that increasing public discussion on drugs encourages youth to look at both pro- and anti-drug websites [46]. Increased exposure to pro-drug websites may indirectly increase overall drug consumption, as was in the case with cigarettes [46].

Holloway recommends social support such as psychological for students with current mental illnesses or alcohol use disorders that put them at higher risk of study drug abuse [4]. This is consistent with previous findings which suggest that helping students feel more comfortable reaching out for help when they need, and ensuring services to accommodate them are available, mitigates harms of consuming study drugs while reducing overall consumption [3, 35, 42, 47].

A drawback of these recommendations on increasing knowledge, promoting factors such as self-efficacy, and accommodating students with special needs is the financial expenses required for them to occur. If financial resources are scarce, postsecondary universities can mitigate expenses through promoting educational and support services they already have or are publicly available to them, such as the case with Western University’s Psychological Services, the Peer Support Centre, the Learning Skills Centre, and the Good2Talk helpline [48]. Using existing services to promote campaigns similar to those they already do, with consideration of reducing harms and promoting well-being for students who use study drugs, may ensure maximum positive outcomes with minimal fee increases.

Another solution to reducing study drug use discussed in Hollway’s (2013) paper focuses on screening and prevention [4, 49]. They describe how some universities in the United Kingdom successfully implement a screening test, called DAST-10, which can help predict a student’s risk of abusing prescription drugs such as Adderall [4, 49]. Students can complete this test upon initial entry to a postsecondary institution [4]. Those at high risk, such as those with previous history of drug use or withdrawal symptoms in the past 12 months, are referred to psychological services for preventative measures [4, 49].

Something to consider is that this strategy might stigmatize students who receive high-risk feedback. The risk of stigmatization is derived from “self-enhancement” and “self-verification” theories in psychology [50]. These theories suggest that people hope to see themselves in a positive manner but might try to see themselves negatively if they are told to believe so, and that negative belief then promotes the negative behavior [50]. People tend to behave in the way that they are described by others [50]. Therefore, the wording of the screening test used plays an important role in its effects on students. If it helps students to feel empowered to be better able to make informed decisions that are best for them, the tool might be very successful in identifying and assisting at-risk groups early. If the test makes students feel vulnerable, weak, or likely to consume these drugs, they may consume them more [50].

One alternative to this is to not share the results with students so that they will not feel more or less at risk than their peers [50]. The information could be used internally, and high-risk groups could receive more information about services offered. An alternative to the potential stigmatization of a particular group could be through sharing social support services to all students at a postsecondary institution, such as Western University currently does, and encouraging students to feel comfortable using them [48]. Therefore, no particular group may feel more vulnerable than another, students will not have to feel pressure sharing results of their survey to peers, and all students will be more educated on the risk-mitigating services available to them.

## Conclusion

Considering underlying risk factors and motivations that encourage postsecondary students to use “study drugs” methylphenidate, dextroamphetamine, and amphetamine are essential to run campaigns that mitigate harms, reduce overall consumption, and provide best outcomes for students who use drugs. These outcomes represent important components of harm reduction, which is becoming increasingly recognized as an important alternative to failing traditional prohibitionist policies against drug use. Study drugs pose serious health and academic risks to students who use them for study purposes and even some individuals who are prescribed them for ADHD. Therefore, it becomes apparent to act quickly in efforts to aid these groups at such an important point in their lives. While many students might use study drugs recreationally without sophisticated underlying issues, understanding at-risk groups that do can help tailor new ways to help students excel in their postsecondary studies and beyond.

## Abbreviations

ADD: Attention deficit disorder
ADHD: Attention deficit hyperactivity disorder
GPA: Grade point average
US: United States
